# Interindividual Variability in Fat Mass Response to a 1-Year Randomized Controlled Trial With Different Exercise Intensities in Type 2 Diabetes: Implications on Glycemic Control and Vascular Function

**DOI:** 10.3389/fphys.2021.698971

**Published:** 2021-09-16

**Authors:** João P. Magalhães, Megan Hetherington-Rauth, Pedro B. Júdice, Inês R. Correia, Gil B. Rosa, Duarte Henriques-Neto, Xavier Melo, Analiza M. Silva, Luís B. Sardinha

**Affiliations:** ^1^Exercise and Health Laboratory, CIPER, Faculdade de Motricidade Humana, Universidade de Lisboa, Lisboa, Portugal; ^2^CIDEFES - Centro de Investigação em Desporto, Educação Física e Exercício e Saúde, Universidade Lusófona, Lisbon, Portugal; ^3^Ginásio Clube Português, GCP Lab, Lisbon, Portugal

**Keywords:** arterial stiffness, Carotid artery intima-media thickness, exercise intervention, peak wave velocity, high-intensity interval training, moderate continuous training

## Abstract

**Purpose**: Little is known about the interindividual variability in fat mass (FM) loss in response to high-intensity interval training (HIIT) and moderate continuous training (MCT) in individuals with type 2 diabetes mellitus (T2DM). Moreover, the impact on health-related outcomes in those who fail to reduce FM is still unclear. The aims of this investigation were (1) to assess if the individuals with T2DM who FM differed across MCT, HIIT, and control groups over a 1-year intervention and (2) to assess the changes on glycemic control and vascular function in the exercising patients who failed to lose FM.

**Methods**: Adults with T2DM were randomized into a 1-year intervention involving a control group (*n*=22), MCT with resistance training (RT; *n*=21), and HIIT with RT (*n*=19). FM was assessed using dual-energy X-ray absorptiometry and a change in total body FM above the typical error was used to categorize FM responders. Glycemic control and vascular stiffness and structure were assessed. A chi-square test and generalized estimating equations were used to model the outcomes.

**Results**: Both MCT (*n*=10) and HIIT (*n*=10) had a similar proportion of individuals who were categorized as high responders for FM, with the percent change in FM on average −5.0±9.6% for the MCT and −6.0±12.1% for the HIIT, which differed from the control group (0.2±7.6%) after a 1-year intervention (*p*<0.05). A time-by-group interaction for carotid artery intima-media thickness (cIMT) (p for interaction=0.042) and lower-limb pulse wave velocity (LL PWV; p for interaction=0.010) between those categorized as low FM responders and the control group. However, an interaction was observed between the high responders for FM loss and controls for both brachial and carotid hemodynamic indices, as well as in cIMT, carotid distensibility coefficient, carotid beta index, and LL PWV (p for interactions <0.05). No interactions were found for glycaemic indices (p for interaction >0.05).

**Conclusion**: Our results suggest that the number of FM responders did not differ between the MCT or HIIT, compared to the control, following a 1-year exercise intervention in individuals with T2DM. However, low responders to FM may still derive reductions in arterial stiffness and structure.

**Clinical Trial Registration**: Comparing Moderate and High-intensity Interval Training Protocols on Biomarkers in Type 2 Diabetes Patients (D2FIT study) – number: NCT03144505 (https://clinicaltrials.gov/ct2/show/NCT03144505).

## Introduction

Obesity is a major contributor to the development of type 2 diabetes (T2DM), with 80% of individuals being classified as obese (Goedecke and Micklesfield, 2014). Several investigations have shown that obesity is associated with insulin resistance and the development of cardiovascular disease in individuals with T2DM (Dube et al., 2011), whereas weight loss, particularly induced by a reduction in fat mass (FM), is a paramount strategy for optimizing glycemic control (Lean et al., 2018) and reducing manifestations of cardiovascular pathology, such as arterial stiffness and structure (Cardoso and Salles, 2016).

Exercise has been shown to be an effective strategy for decreasing body fat although the type, frequency, duration, and intensity most effective for reducing adiposity remain debated in individuals with T2DM (Dube et al., 2011; De Nardi et al., 2018). In short to medium-term interventions, high-intensity interval training (HIIT) has been proposed as a time efficient training method that may induce greater reductions in FM when compared to moderate-intensity continuous training (MCT) in individuals with T2DM (Liu et al., 2019). However, all of these exercised-based interventions rely on group mean effects for FM loss, which provides no information about the interindividual variability of FM changes in response to HIIT and MCT in individuals with T2DM (Chrzanowski-Smith et al., 2020). A previous investigation comparing continuous aerobic training at different intensities during a 24-week intervention period showed that in obese adults, there was a higher number of individuals in the higher intensity exercise group achieving a clinically important reduction in visceral adipose tissue (<0.28kg), when compared to those in the moderate-intensity group (Brennan et al., 2020a). Whether HIIT affects the proportion of individuals with T2DM who are likely to achieve a clinically meaningful FM reduction following a long-term intervention is unknown.

Regardless of the alteration in the exercise characteristics, there still remains a portion of individuals who do not achieve clinical meaningful FM loss (Stephens and Sparks, 2015). Nevertheless, irrespective of reductions in FM, heterogeneity in the effects of exercise on cardiometabolic outcomes exist, such that improvements in glycemic control and vascular function have been found independent of FM (Tanaka et al., 2000; Gaesser et al., 2011; Hawkins et al., 2014). Although FM plays a major role in the pathophysiology of T2DM, exercise can work through other pathways to induce beneficial changes in two of the most impacted systems of this disease, being glycemic control and vascular function. In fact, we recently have shown that regardless of the cardiorespiratory fitness (CRF) response to 1-year of exercise, favorable changes in vascular structure and function were found (Hetherington-Rauth et al., 2020a). On this matter, no longitudinal randomized controlled trial (RCT) with different exercise intensities using an ecological approach has yet analyzed the glycemic and vascular benefits in patients who do not achieve meaningful fat loss.

Therefore, the aims of this investigation were 2-fold: (1) to compare the response to FM loss following 1year of MCT or HIIT in individuals with T2DM and (2) to assess whether individuals who failed to attain exercise-derived clinically meaningful reductions in FM still improved their cardiovascular risk profile by improving glycemic control and reducing local and regional manifestations of cardiovascular pathology, such as arterial stiffness and structure.

## Materials and Methods

### Subjects

The current investigation assessed individuals with T2DM who took part in a 1-year exercise RCT (D2FIT study) with three distinct arms: a non-exercise control group, a MCT with resistance training (RT) group, and a HIIT with RT group (ClinicalTrials.gov registration no. NCT03144505). The randomization proceeded with a 1:1:1 allocation ratio between the three intervention groups by a researcher external to the D2FIT study and blinded to the enrolment process, using computer-generated list of random numbers. The study design and methodology of D2FIT study have been previously published (Magalhaes et al., 2019a).

The primary outcome of D2FIT study concerned changes in glycated hemoglobin (HbA1c), which was assessed at baseline and at the end of the intervention period (i.e., 1year). Participants were recruited in Lisbon, Portugal between February 2014 and July 2016. Eligible criteria included as: adults previously diagnosed with T2DM (American Diabetes, A, 2020); aged 30–75years; no major micro-and macrovascular complication from T2DM; body mass index <48kg/m^2^; and no physical limitation preventing individuals from exercising. Power and sample size calculations (G-Power, Version 3.1.3, Düsseldorf, Germany) were based on a predicted HbA1c change of 0.66units with an SD of 1.2units, *α*=0.05, 1-β=0.80, and an expected dropout rate of 10% (Boule et al., 2001). A total of 80 individuals were selected and randomized, however, for the current analysis only participants who completed the 1-year investigation (*n*=62) were considered.

Written informed consent was obtained from all participants prior to screening. The D2FIT study protocol was reviewed and approved by the Ethics Committee of the Portuguese Diabetes Association (approval number: 07/17/2013).

### Exercise Intervention

Exercise prescription and session time were standardized based on physical activity (PA) guidelines (U.S. Department of Health and Human Services, 2008) to achieve a weekly target of 41.84kJ/kg (10kcal/kg) for both the MCT and HIIT group.

Throughout the intervention, individuals from both groups received a structured periodization of the exercise program with an individualized and supervised intensity of training based upon heart rate reserve (HRR). A full detailed description of the periodization protocol can be found elsewhere (Magalhaes et al., 2019a).

Participants in the HIIT with RT (*n*=19) and MCT with RT groups (*n*=21) exercised 3days per week. The MCT group performed continuous cycling on a cycle ergometer (Monark Ergometric 828e, Vansbro, Sweden) at 40–60% of HRR throughout the intervention. The HIIT group performed 1min of cycling at 90% of HRR, followed by a 1min rest period at 40–60% of HRR (1:1 exercise:rest ratio). Following the aerobic training component, participants from both groups underwent a specific RT including one set of 10–12 repetitions of upper- and lower-limb exercises. The intensity of all trainings was monitored using a heart rate monitor (Polar T-31, Bethpage, NY, United States) worn on the participant’s chest.

The control group had an initial orientation session with standard counseling regarding general PA guidelines, with an additional session every month where thematic sessions were held in order to discuss topics, such as nutrition or PA as a retention strategy.

### Anthropometry and Body Composition

Participants weight and height were measured according to standardized procedures (Lohman et al., 1988).

Total FM was estimated using dual-energy X-ray absorptiometry (DXA; Hologic Explorer-W, Waltham, United States). Following the protocol for DXA described by the manufacturer, a laboratory technician positioned the participants, performed the scans, and executed the analyses according to the operator’s manual. The %CV in our laboratory is 1.7 for FM and 0.8 for lean mass (Santos et al., 2013).

### Hemodynamic Indices

Brachial systolic blood pressure and diastolic blood pressure (bDBP) were measured using an automated oscillometric cuff (HEM-907-E; Omron, Tokyo, Japan) following the participant lying 15min in the supine position. Carotid systolic blood pressure (cSBP) and carotid diastolic blood pressure were measured using ultrasound scanner equipped with a linear 13MHz probe (MyLab One, Esaote, Italy). The mean arterial pressure (MAP) was calculated using the formula: MAP=DBP + [1/3(SBP−DBP)].

### Local Carotid Artery Intima-Media Thickness

Carotid artery intima-media thickness (cIMT) was measured on the far wall of the right carotid artery using an ultrasound scanner equipped with a linear 13MHz probe (MyLab One, Esaote, Italy; Hoeks et al., 1997). Distension curves were acquired within a segment of the carotid artery ~1cm before the flow divider.

### Carotid Arterial Stiffness Indices

After 15min in a supine position, an ultrasound scanner equipped with a linear 13MHz probe (MyLab One) was placed ~1cm before the carotid artery bifurcation on the right side of the body and used to calculate pulse wave velocity (PWV; m/s), carotid distensibility coefficient (DC; 1/Kpa), and stiffness index β. A detailed description can be found elsewhere (Hetherington-Rauth et al., 2020b).

### Regional PWV

The distance between the carotid and femoral and radial and distal posterior tibial arteries were measured using applanation tonometry and values were directly inserted into the Complior Analyse software (ALAM Medical, Paris, France). PWV values obtained from measurements of the carotid to femoral artery, carotid to radial artery, and carotid to distal posterior tibial artery were taken as indices of aortic and peripheral arterial stiffness for upper (UL) and lower limbs (LL), respectively. A detailed description can be found elsewhere (Hetherington-Rauth et al., 2020b).

### Laboratory Measurements

Fasting blood samples were collected for the assessment of glucose, insulin, and HbA1c before a mixed meal tolerance test and 30 and 120min after beginning of meal consumption (two bottles of boost complete nutritional drink) for glucose and insulin. Samples were drawn into chilled, heparinized tubes and centrifuged rapidly to avoid glycolysis. Plasma glucose was measured by photometry (auto analyzer Olympus AU640, Beckman Coulter). Plasma insulin was analyzed using electrochemiluminescence immunoassays (Liaison, Diasorin). HbA1c was analyzed by immunoassay (auto analyzer Hb9210 Premier A. Menarini Diagnostics). The homeostasis model assessment (HOMA) variables were estimated using the HOMA2 calculator.^1^

### Physical Activity

Both the control and the exercise groups wore an accelerometer every 3months to access their physical activity and sedentary behavior (ActiGraph, GT3X+ model; Fort Walton Beach, FL, United States). Participants were asked to wear the accelerometer on the right hip, close to the iliac crest, for 7 consecutive days. The device activation, download, and processing were performed using the software, Actilife (v.6.9.1; ActiGraph). Data were recorded using the raw mode with a 100Hz frequency and posteriorly downloaded into 15s epochs. Troiano et al. cut-points and validation criteria were used for data analysis (Troiano et al., 2008).

### Identifying Individual Exercise Fat Mass Responders

Currently, there are no accepted guidelines for the percent of FM loss considered to be clinically meaningful (Brennan et al., 2019). Therefore, we considered someone who had a FM loss greater than the typical error (TE) as clinically meaningful. The TE was calculated from the SD of the differences in FM over 1year in the control group (TE=SD_diff_/√2), as described by Hopkins (2000) and used by others (Walsh et al., 2020; Brennan et al., 2020a). The TE represents the technical error of measurement as well as the within-subject variability caused by changes in behavioral/environmental factors across an intervention (Bonafiglia et al., 2018). The TE for FM in our study was 1.73kg (i.e., ~−6% FM from baseline). Hence, any individual with a FM loss >1.73kg was considered to be a high responder and individuals with FM ≤1.73kg were considered low responders.

### Statistical Analysis

Descriptive statistics, including measures of central tendency (mean) and variability (SD) for normally distributed variables and median (interquartile range) for skewed variables, were used to describe baseline descriptive characteristics. Depending on normality and variable type, a one-way ANOVA with Bonferroni adjustment for multiple comparisons, Kruskal–Wallis test, *χ*^2^ test, or Fisher’s exact test were used to compare baseline measures between groups.

Differences in the proportion of individuals in the control, MCT, and HIIT groups who reduced FM after 3, 6, and 12months of intervention were assessed using a chi-square test.

Generalized estimating equations were used to assess outcomes indicative of glycaemic control and vascular structure and function, while adjusting for sex, baseline moderate-to-vigorous PA (MVPA), number of training sessions completed, and percent changes in MAP (only in models assessing differences in arterial stiffness and structure indices). A linear distribution with an identity link function for the response was assumed and an autoregressive model with a robust estimator was used for the working correlation matrix and covariance matrix, respectively. Finally, the maximum likelihood estimate was set to the data to calculate the parameter estimation and the lowest value for the goodness of fit was used for comparisons between models.

A Bonferroni post-hoc test was used to estimate the between- and within-group effects. A linear distribution for the response was assumed and an autoregressive correlation matrix was set to the data.

A value of p of <0.05 was considered statistically significant. Data analyses were performed using IBM SPSS Statistics version 22.0 (SPSS, Chicago, IL, United States) and STATA version 13.1 (StataCorp, College Station, TX, United States).

## Results

Table 1 describes baseline values between high responders (*n*=20; ∆FM >TE), low responders (*n*=20; ∆FM ≤TE), and the control group (*n*=22). Out of the 62 participants (45% female), 53% were categorized as obese, and 31% were overweight. At baseline, we found no differences (*p*≥0.05) between the low and high responders, except for MVPA, in which high responders had higher values when compared to the controls (*p*<0.05). On the other hand, and considering the Canadian physical activity guidelines (Ross et al., 2020), all groups spent a considerable amount of time in sedentary pursuits as shown by accelerometer data and time spent watching TV.

**Table 1:** Baseline descriptive characteristics of control group and exercise groups who either reduced or did not reduce their total body FM.

	Control (*n* =22)	Low responders (*n*=20)	High responders (*n*=20)	Value of p
	Mean (SD)	Mean (SD)	Mean (SD)	
Age (years)	60.8 ± 7.5	57.5 ± 8.9	59.6 ± 7.0	0.39
Gender (F:M)	11:11	8:12	9:11	0.81
Weight (kg)	84.6 ± 15.4	82.1 ± 18.1	80.5 ± 11.3	0.67
Height (cm)	164.5 ± 9.5	165.0 ± 8.8	163.9 ± 8.1	0.93
Body mass index (kg/m^2^)	31.7 ± 4.7	30.5 ± 6.0	30.6 ± 5.2	0.73
Time from DM dx	5.9 ± 5.4	7.8 ± 4.9	7.5 ± 5.2	0.44
Hypertensive medication (%)	54.5	45.0	50.0	0.82
Oral hypoglycemic medication	95.5	95.0	90.0	0.83
% Trainings completed (%)	NA	74.2 ± 20.7	77.5 ± 22.4	0.63
MCT (n): HIIT (n)	NA	11:9	10:10	0.75
Baseline MVPA (min/d)^a^	22.1 ± 16.0	31.0 ± 18.6	46.9 ± 30.8*	**0.003**
Sedentary time (min/d)^a^	570.1 ± 147.4	603.5 ± 67.2	570.3 ± 86.6	0.527
TV viewing time (min/d)	199.3 ± 141.2	195.3 ± 108.7	186.8 ± 143.1	0.952
HbAIC (%)^a^	6.9 ± 1.1	7.3 ± 1.4	6.9 ± 1.1	0.54
HbAIC (mmol/L)^a^	51.7 ± 11.8	55.9 ± 15.7	52.1 ± 12.3	0.54
Fasting glucose (mg/dL)^a^	8.0 ± 1.8	9.8 ± 3.7	8.5 ± 3.6	0.18
VO_2peak_ (ml/min/kg)	25.2 ± 5.6	25.5 ± 5.1	25.7 ± 6.1	0.95
bMAP (mmHg)	91.6 ± 9.0	96.2 ± 13.0	99.3 ± 10.5	0.08
Total body FM (kg)	29.9 ± 6.8	28.1 ± 9.4	28.2 ± 8.5	0.73
Total body % FM (%)	35.5 ± 6.3	33.9 ± 6.7	34.7 ± 7.7	0.73
Total body lean soft tissue (kg)	52.3 ± 11.4	51.6 ± 11.0	49.9 ± 7.4	0.73
bSBP (mmHg)	127.4 ± 17.3	133.3 ± 18.5	136.1 ± 16.7	0.28
bDBP (mmHg)	73.8 ± 6.7	78.0 ± 11.7	81.0 ± 9.3	0.06

DM, diabetes mellitus; bSBP, brachial systolic blood pressure; bDBP, brachial diastolic blood pressure; FM, fat mass; HbAIC, glycated hemoglobin; HIIT, high-intensity interval training; MAP, mean arterial pressure; MCT, moderate continuous training.aMedian (interquartile range). *significantly different from control, p<0.05.

At the end of the 1-year intervention, 20 individuals in the exercise groups (10 from MCT and 10 from HIIT) decreased their FM above the TE threshold, compared with only two individuals in the control group (*p*>0.05; Table 2).

**Table 2:** Proportion of individuals in experimental groups who reduced body fat mass at different time points.

	Control	MCT	HIIT	Value of p
High responders, n (%)				
3months	3 (14.3)	8 (38.0)	5 (26.3)	0.22
6months	4 (19.1)	8 (38.1)	6 (35.3)	0.41
12months	2 (9.1)	10 (47.6)	10 (52.6)	0.004

*MCT, moderate continuous training; HIIT, high-intensity interval training; FM, fat mass; and TE, technical error. Values presented as absolute and percentage*.

**Figure 1:**
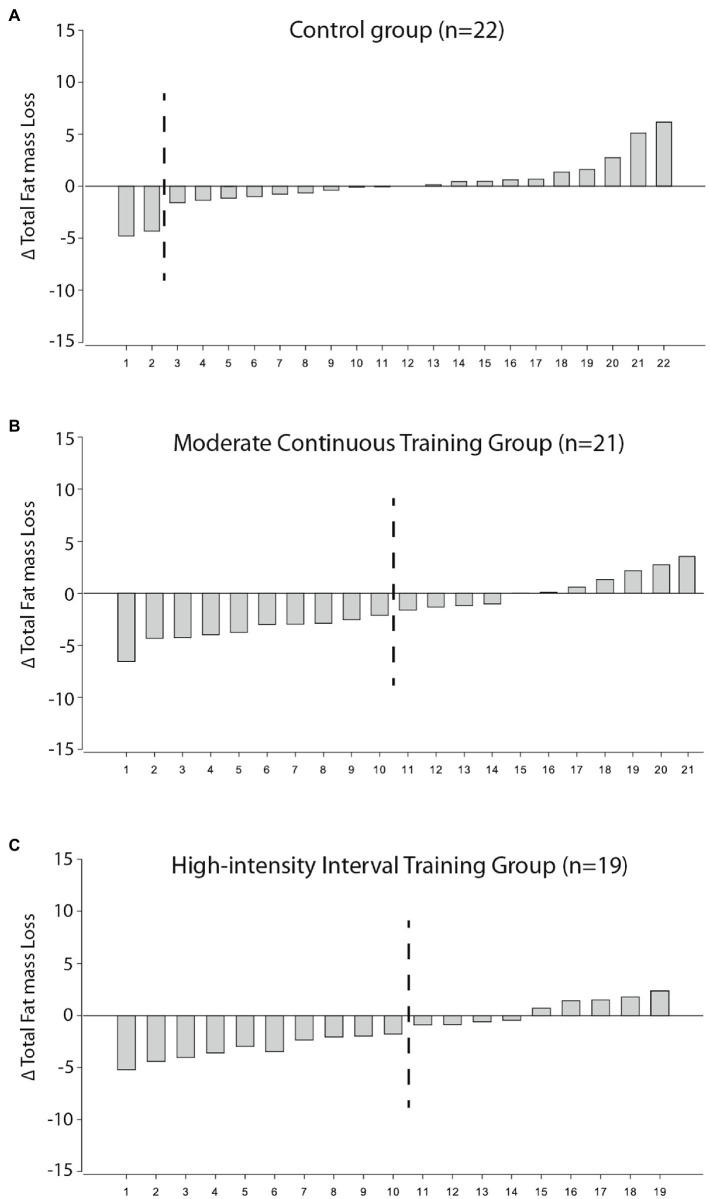
Individual response changes for total FM loss in the control **(A)**, MCT **(B)**, and HIIT group **(C)** according to 90% CI SWC cutoffs. Those on the left of the dashed black line are the participants who were considered high responders according to ΔFM loss>TE. TE, typical error.

Figure 1 shows the individual changes in FM loss for each participant in the control (Figure 1A), MCT (Figure 1B), and HIIT groups (Figure 1C) after 1-year relative to the TE cutoffs.

Table 3 depicts the within- and between-group changes in glycemic control, hemodynamic indices, and indices of vascular stiffness and structure in control and FM responder groups. The high FM responders had favorable changes in vascular structure and stiffness indices as indicated by the time-by-group interaction observed between the cIMT (p for interaction <0.001), carotid DC (p for interaction=0.016), beta stiffness index (p for interaction=0.035), carotid PWV (p for interaction=0.038), and LL PWV (p for interaction=0.021) with the control group. Favorable changes were also observed on hemodynamic indices between the high FM responder group and controls (p for interactions <0.05), except for cSBP and carotid mean arterial pressure (cMAP; p for interactions >0.05). Moreover, for the hemodynamic indices, similar interactions were observed between the high FM responder and low FM responder groups (p for interactions <0.05). Although no interactions in hemodynamic indices were observed between the low FM responders and the controls (*p*>0.05), there still was a time-by-group interaction between the low FM responders and the controls in vascular structure (cIMT: p for interaction=0.042) and vascular stiffness (LL PWV: p for interaction=0.010). No interactions were observed between the high and low FM responders and the controls for glycemic outcomes (p for interactions >0.05).

**Table 3:** Within- and between-group changes in vascular health of control and low and high FM responders’ groups.

*Outcome*	Control (*n* =22)		Low FM Responders (*n*=20)		High FM Responders (*n*=20)		Low FM Responders * Control	High FM Responders * Control	High FM Responders * Low FM Responders
	Baseline	12months	Baseline	12months	Baseline	12months	**β** (95% CI)	**β** (95% CI)	**β** (95% CI)
*Glycemic indices*									
Fasting glucose (mmol/mol)	7.3 ± 1.8	7.8 ± 1.8	9.8 ± 3.7	9.9 ± 4.6	8.5 ± 3.6	8.2 ± 3.6	0.03 (−0.08, 0.13)	−0.01 (−0.11, 0.10)	0.03 (−0.09, 0.15)
IAUC Glucose	7.3 ± 3.7	7.7 ± 3.6	7.9 ± 3.8	9.0 ± 3.9	7.6 ± 4.2	7.8 ± 3.7	0.05 (−0.13, 0.24)	−0.01 (−0.18, 0.15)	0.07 (−0.13, 0.27)
IAUC Insulin	751.9 ± 546.5	703.6 ± 647.7	493.6 ± 301.9	575.1 ± 493.0	443.1 ± 388.3	502.5 ± 502.8	15.91 (−4.78, 36.59)	17.83 (−3.65, 39.30)	−1.20 (−17.55,13.71)
HOMA2-IR	2.4 ± 1.6	2.4 ± 1.7	2.2 ± 1.4	2.1 ± 1.8	1.5 ± 0.6	1.5 ± 0.7	−0.001 (−0.07, 0.07)	−0.01 (−0.06, 0.07)	−0.03 (−0.05, 0.05)
HgAIC (mmol/mol)	51.7 ± 11.8	54.7 ± 11.2	55.9 ± 15.7	58.9 ± 16.5	52.1 ± 12.3	52.9 ± 14.8	0.13 (−0.36, 0.63)	−0.07 (−0.56, 0.45)	0.19 (−0.22, 0.60)
*Hemodynamic índices*									
bSBP (mmHg)	127.4 ± 17.3	131.5 ± 19.2	133.3 ± 18.5	138.3 ± 19.6	136.1 ± 16.7	129.6 ± 15.6	0.10 (−0.62, 0.83)	**−0.90 (−1.60, −0.20)** *	**1.01 (0.34, 1.67)** *
bDBP (mmHg)	73.8 ± 6.7	74.0 ± 11.0	78.0 ± 11.7	78.4 ± 7.1	**81.0 ± 9.3**	**75.6 ± 8.2**†	0.05 (−0.41, 0.51)	**−0.48 (−0.90, −0.07)** *	**0.53 (0.07, 0.99)** *
bMAP (mmHg)	91.5 ± 9.0	93.0 ± 11.8	96.2 ± 13.0	98.1 ± 9.6	99.3 ± 10.5	93.5 ± 9.6	0.06 (−0.45, 0.57)	**−0.63 (−1.09, −0.17)** *	**0.69 (0.20, 1.19)** *
cSBP (mmHg)	121.9 ± 25.3	121.2 ± 19.3	121.0 ± 20.0	127.6 ± 21.1	122.7 ± 14.0	117.6 ± 15.2	0.61 (−0.44, 1.66)	−0.40 (−1.33, 0.52)	**1.01 (0.31, 1.71)** *
cDBP (mmHg)	73.8 ± 6.7	74.0 ± 11.0	78.0 ± 11.7	78.4 ± 7.1	81.0 ± 9.3	75.6 ± 8.2	0.05 (−0.41, 0.51)	**−0.48 (−0.90, −0.07)** *	**0.54 (0.07, 0.99)** *
cMAP (mmHg)	89.8 ± 11.8	89.8 ± 12.2	92.3 ± 13.4	98.4 ± 10.4	94.9 ± 9.8	89.6 ± 9.5	0.23 (−0.36, 0.82)	−0.47 (−0.96, 0.02)	**0.70 (0.20, 1.20)** *
*Vascular stiffness and structure indices*									
Carotid IMT (mm)	**714.9 ± 130.7**	**751.2 ± 119.4** *	740.8 ± 203.9	723.3 ± 156.7	**704.2 ± 139.7**	**673.9 ± 116.6** *	**−4.34 (−8.53, −0.15)** *	**−5.40 (−8.27, −2.53)** *	1.01 (−3.35, 5.47)
Carotid PWV (m/s)	**7.5 ± 1.3**	**8.1 ± 1.9**†	7.6 ± 1.5	7.9 ± 1.7	7.7 ± 1.3	7.6 ± 1.5	−0.03 (−0.07, 0.02)	**−0.05 (−0.11, −0.01)** *	003 (−0.02, 0.07)
Carotid DC (1/Kpa)	**1.8 ± 0.6e** ^**−2**^	**1.5 ± 0.6e**^-2*^†	0.02 ± 0.8e^−2^	0.02 ± 0.8e^−2^	0.02 ± 0.7e^−2^	0.02 ± 0.8e^−2^	1.80e^−4^ (−4.91e1^−5^, 4.09e^−5^)	3.47e^−4^ (6.48e^−5^, 6.28e^−4^)*	−1.67e^−5^ (−4.45e^−5^, 1.11e^−5^)
Carotid β	**11.9 ± 4.5**	**14.2 ± 6.8**†	**11.9 ± 4.9**	**13.5 ± 3.4**†	12.2 ± 4.2	12.1 ± 4.2	−0.06 (−0.23, 0.10)	**−0.19 (−0.34, −0.04)** *	0.13 (−0.04, 0.29)
Aortic PWV (m/s)	13.1 ± 4.8	14.0 ± 4.3	13.0 ± 3.0	13.5 ± 3.4	13.3 ± 4.2	114.5 ± 4.5	−0.03 (−0.17, 0.11)	0.02 (−0.11, 0.15)	−0.05 (−0.17, 0.07)
UL PWV (m/s)	9.2 ± 1.9	9.3 ± 1.5	9.5 ± 1.6	9.0 ± 1.7	10.0 ± 2.2	9.2 ± 2.4	−0.05 (−0.16, 0.06)	−0.08 (−0.20, 0.04)	0.03 (−0.09, 0.15)
LL PWV (m/s)	**9.0 ± 1.8**	**10.3 ± 1.7**†	9.9 ± 2.1	9.6 ± 2.0	10.1 ± 2.4	9.7 ± 2.6	**−0.13 (−0.24, −0.03)** *	**−0.15 (−0.27, −0.02)** *	−0.01 (−0.09, 0.12)
Total body FM (kg)	29.9 ± 6.8	30.1 ± 7.6	28.1 ± 9.4	28.6 ± 9.5	**28.2 ± 8.5**	**24.8 ± 8.4**†	−0.04 (−0.06, 0.14)	**−0.26 (−0.36, −0.16)** *	**0.30 (0.22, 0.38)** *

*β=unstandardized β adjusted for sex, total number of trainings completed, and baseline MVPA and age; unstandardized β for carotid structure and stiffness and regional arterial stiffness indices additionally adjusted for percent change in MAP*

*IAUC, incremental area under the curve; FM, fat mass; CRF, cardiorespiratory fitness; bSBP, brachial systolic blood pressure; cSBP, carotid systolic blood pressure; bDBP, brachial diastolic blood pressure; cDBP, carotid diastolic blood pressure; MAP, mean arterial pressure; IMT, intima-media thickness; PWV, pulse wave velocity; DC, distensibility coefficient; UL, upper limb; and LL, lower limb*.

* *Between-group changes significant at p<0.05; † Within-group changes significant at p<0.05.*

## Discussion

To the best of our knowledge, this is the first investigation to address the response rate to changes in FM following a long-term intervention with both MCT and HIIT in individuals with T2DM. Following a 1year of exercise, we found that the proportion of individuals who attained meaningful changes in FM (high responders) differed between the exercise and the control groups, but no differences were found between the MCT and HIIT. Moreover, those considered low responders still had favorable changes on vascular structural and stiffness indices, such as cIMT and LL PWV. Despite the benefits observed in low responders, individuals with higher FM losses had superior benefits, not only on cIMT and LL PWV, but also on other stiffness indices and hemodynamic outcomes, which may have favorable implications on the progression of diabetes related macrovascular complications.

With a similar approach, in an obese population, Brennan et al. (2020a) aimed to determine the effect of different exercise intensities on the proportion of individuals who had meaningful reductions in total and abdominal adipose tissue (i.e., responders) following 24weeks of intervention. Their results suggested that increasing exercise amount and/or intensity may increase the proportion of individuals who achieve clinically meaningful visceral adipose tissue reductions. In our study, we observed a difference in the proportion of high responders in both the MCT and the HIIT group when compared to the control; however, no differences were found between exercise intensities. The lack of differences between our exercise intensities (i.e., MCT *vs.* HIIT), as opposed to those observed in Brennan et al. (2020a), may be due to the longer length of our intervention period (1year *vs.* 24weeks) and the intensity of our exercise protocol (HIIT at 90% HRR *vs.* continuous vigorous exercise at >75% VO_2max_), both of which may have led to higher experienced physiological and psychological fatigue by the HIIT group toward the end of our intervention, impairing their overall 1-year exercise outcomes compared to MCT. In fact, the population differences between studies (T2DM *vs.* obese adults) may also explain why HIIT did not have a higher proportion of responders compared to the MCT, given that individuals with T2DM are known to have a reduced peak workload capacity, peak oxygen assumption, oxygen pulse, and ventilatory efficiency, thus potentially inhibiting the additional effects of a more demanding exercise protocol (e.g., HIIT; Nesti et al., 2020).

When assessing the number of high responders in each group over the length of the intervention, we did not find differences in either of the exercise groups and the controls at the 3- and 6-month mark. This is likely due to the fact that, for ethical reasons, our control group had monthly sessions, where topics of nutrition and PA were discussed, hence increasing their odds of being categorized as high responders. Nevertheless, at the 12-month mark, there were differences between the proportion of high responders in both the MCT and HIIT group compared to control, which is partly in line with our previously published main outcomes, where a time-by-group interaction for total FM loss was observed at the 1-year mark in the MCT group, but not for the HIIT group (Magalhaes et al., 2019a). These results put in perspective the importance of looking not only at the mean effects of an exercise intervention, but also to the added value of exploring the individual responses to exercise.

We have previously reported the main results of the D2FIT study, where glycemic and vascular health outcomes varied depending on the exercise intensity (Magalhaes et al., 2019a,b). As part of our secondary aim, we further explored whether individuals classified as low responders for FM loss could still benefit from the exercise intervention. We observed no time-by-group interaction for all of the glycemic control outcomes when comparing the high and low responders against the control group. Conversely, we found that being categorized as a low responder did not preclude individuals from having improvements in vascular and stiffness indices (i.e., cIMT and LL PWV) when compared to the controls. Despite benefits in vascular function being independent of FM loss (Tanaka et al., 2000; Hawkins et al., 2014), those classified as high responders had superior benefits in vascular health, as observed by the time-by-group interaction on cIMT, carotid DC, beta stiffness index, carotid PWV, and LL PWV, in addition to improvements in blood pressure parameters when compared to controls. Indeed, a positive relationship between obesity and blood pressure and risk for hypertension has been thoroughly documented (Hubert et al., 1983; Fogari et al., 2010). In our investigation, only the high responders had a time effect on their DBP, with a reduction of ~6mmHg, which is noteworthy given the estimated 15–27% reduced incidence of CVD with a decrease of 3.0mmHg in DBP (Appel et al., 1997). Plausible mechanisms, such as improvements in the sympathetic nervous system and the renin–angiotensin–aldosterone system, may explain the favorable changes in hemodynamic outcomes observed in FM high responders (Kurukulasuriya et al., 2011; DeMarco et al., 2014).

Although improvements in glycemic control with exercise are a main driving factor for the favorable changes in structural and functional vascular indices (MacDonald et al., 2020), neither the low responders or high responders improved their glycemic control when compared to the control group. Thus, other mechanisms may be responsible for the actual improvements observed in vascular parameters in both groups. Indeed, the action of exercise itself can improve vascular function through increasing cardiac output, hence affecting systemic blood flow and impacting endothelial shear stress, which increases the forces exerted on the arterial wall that lead to the production of nitric oxide (Green et al., 2017).

Lastly, similar to the results of this study, we previously observed a similar pattern when considering CRF response to this same exercise intervention, where a low responder to CRF was not precluded from improvements in vascular health (Hetherington-Rauth et al., 2020a). Given that both CRF and FM are two of the most used clinical measurements indicative of the successfulness of an exercise intervention, our current results extend those previously published on CRF by showing that vascular benefits can still be obtainable with exercise in individuals with T2DM who lack reductions in total FM. Nevertheless, individuals classified as high responders for FM loss seem to have superior vascular health benefits, compared to low responders, which was not observed when classifying responders based on CRF.

In an era of personalized lifestyle-based medicine and with an increase in the number of interventions focused on the interindividual variability of several cardiometabolic risk factors in response to exercise (Alvarez et al., 2017; Solomon, 2018; Brennan et al., 2019; Ross et al., 2019; Hetherington-Rauth et al., 2020a; Brennan et al., 2020b), there is a clear need to have a control group to differentiate the inevitable within-subject random variability due to biological error and the technical error of measurement from the variability resulting from the exercise intervention. A major strength of the D2FIT study was its long-term RCT design in individuals with T2DM, which follows the recent consensus statement recommendations, highlighting the importance of using a control group (Ross et al., 2019; Padilla et al., 2021).

The present investigation is not without limitations. The baseline differences observed for the time spent in MVPA may have contributed to the variability observed between the responders and non-responders; however, the results remained unchanged after adjusting for baseline MVPA levels. Due to the nature of this secondary analysis, the originally isoenergetic MCT and HIIT groups were rearranged based on their response rate for FM loss, which could have led to an un-matched exercise volume between the low and high responders. Despite these changes, the proportion of participants from the MCT and HIIT groups, as well as the percent trainings completed, was similar between the low and high responders.

There is still a potential risk to misclassify responders using the TE, which could be reduced by incorporating the 90% confidence intervals on top of the TE, as suggested by Bonafiglia et al. (2018). Nevertheless, for our investigation, this approach would be too conservative, given our high TE derived from the different FM losses observed in the control group, which were likely due to the length and the design of our study (for ethical reasons and participant retention, the control group had monthly sessions on how to improve several aspects of diabetes care, including exercise and diet). However, even when using the TE approach, we still observed variability in the responses to exercise training for FM loss at the 1-year mark with an average percent FM loss of ~13% in the high FM responder group. In fact, this is considerably large considering that in general a 5% loss of body weight is considered to be clinically meaningful for the improvement of cardiometabolic risk factors (Jensen et al., 2014).

In conclusion, a 1-year exercise intervention of either a MCT or HIIT protocol combined with RT had a superior proportion of T2DM individuals who were classified as high responders when compared to the control group. Moreover, individuals who did not improve their body FM following the 1-year intervention still had beneficial adaptations on vascular structure and stiffness indices. Still, high responders to FM loss had additional improvements in vascular health and blood pressure. Practitioners should not overlook the other benefits on vascular health that can arise from exercise in those who are classified as low responders to FM loss. Nevertheless, FM loss is still an important outcome of exercise interventions to further reduce the progression of CVD in individuals with T2DM.

## Data Availability Statement

The datasets presented in this article are not readily available because data sharing was not included in the Ethics Committee proposal. Requests to access the datasets should be directed to lbsardinha@fmh.ulisboa.pt.

## Ethics Statement

The studies involving human participants were reviewed and approved by the Ethics Committee of the Portuguese Diabetes Association (approval number: 07/17/2013). The patients/participants provided their written informed consent to participate in this study.

## Author Contributions

LS and JM contributed to the conception and design of the study. JM, PJ, IC, GR, DH-N, and XM were responsible for data collection and acquisition. MH-R and EC were responsible for data analysis and interpretation. JM and MH-R drafted the manuscript. AS, EC, LS, JM, PJ, IC, DH-N, GR, and XM contributed to reviewing and editing the manuscript. All authors contributed to the article and approved the submitted version.

## Funding

This work was supported by the fellowships from the Portuguese Foundation for Science and Technology (grant to JM: SFRH/BD/85742/2012; IC: SFRH/BD/149394/2019; and GR: 2020.07856.BD). This work is also financed by a national grant through the Fundação para a Ciência e Tecnologia (FCT) within the unit I&D 447 (UIDB/00447/2020).

## Conflict of Interest

The authors declare that the research was conducted in the absence of any commercial or financial relationships that could be construed as a potential conflict of interest.

## Publisher’s Note

All claims expressed in this article are solely those of the authors and do not necessarily represent those of their affiliated organizations, or those of the publisher, the editors and the reviewers. Any product that may be evaluated in this article, or claim that may be made by its manufacturer, is not guaranteed or endorsed by the publisher.

## Footnotes

^1^ https://www.dtu.ox.ac.uk/homacalculator/

## Abbreviations

Body mass index: BMI
Brachial systolic blood pressure: SBP
Cardiorespiratory fitness: CRF
Carotid mean arterial pressure: cmap
Carotid systolic blood pressure: csbp
Diastolic blood pressure blood pressure: DBP
Dual-energy X-ray absorptiometry: DXA
Fat mass: FM
Glycated hemoglobin: hba1c
Heart rate reserve: HRR
High-intensity interval training: HIIT
Homeostasis model assessment: HOMA
Individual response standard deviation: SD_IR_
Intima-media thickness: IMT
Mean arterial pressure: MAP
Moderate-intensity continuous training: MCT
Physical activity: PA
Pulse wave velocity: PWV
